# Longitudinal trajectory of disability in community-dwelling older adults: An observational cohort study in South Korea

**DOI:** 10.1186/s12877-020-01834-y

**Published:** 2020-10-28

**Authors:** Hae Reong Kim, Heayon Lee, Yoonje Seong, Eunju Lee, Hee-Won Jung, Yu Rang Park, Il-Young Jang

**Affiliations:** 1 Department of Biomedical Systems Informatics, Yonsei University College of Medicine, Seoul, South Korea; 2 Division of Geriatrics, Department of Internal Medicine, Asan Medical Center, University of Ulsan College of Medicine, Seoul, South Korea; 3 Division of Pulmonary, Critical Care and Sleep Medicine, Department of Internal Medicine, Eunpyeong St. Mary’s Hospital, College of Medicine, The Catholic University of Korea, Seoul, South Korea; 4 Yonsei University College of Medicine, Seoul, South Korea; 5 Pyeongchang Health Center & Country Hospital, Gangwon-do, South Korea

**Keywords:** Aged, Disability, Geriatric assessment, Patient-Centered Care, Quality of life, Trajectory

## Abstract

**Background:**

Disability, which is considered a health-related condition, increases care demands and socioeconomic burdens for both families and communities. To confirm the trend of dynamic longitudinal changes in disability, this study aims to explore how disability is divided by the trajectory method, which deals with time-sequenced data. Additionally, this study examines the differences in demographics, geriatric conditions, and time spent at home among the trajectory groups in community-dwelling older adults. Home time is defined as the period during which the patient was not in a hospital or health care facility during their lifetime.

**Methods:**

Records of 786 community-dwelling older participants were analyzed from the Aging Study of PyeongChang Rural Area, a population-based cohort study that took place over three years. Using 7 domains of activities of daily living and 10 domains of instrumental activities of daily living, participants were grouped into no dependency (0 disabled domain), mild (1 disabled domain), and severe (2 or more disabled domains) disability groups. The longitudinal trajectory group of disability was calculated as a trajectory method. Three distinct trajectory groups were calculated over time: a relatively-stable group (78.5%; *n* = 617), a gradually-aggravated group (16.0%; *n* = 126), and a rapidly-deteriorated group (5.5%; *n* = 43).

**Results:**

The average age of 786 participants was 73.3 years (SD: 5.8), and the percentage of female was 52.7%. It was found that 78.5% of patients showed relatively no dependence and 5.5% of older adults in a rural area showed severe dependence. Through applying the trajectory method, it was shown that the Short Physical Performance Battery (SPPB) score was 10.2 points in the relatively-stable group and 3.1 points in the rapidly-deteriorating group by the 3rd year. Additionally, by the trajectory method, the rate of decrease in home time was 3.33% in the rapidly-deteriorated group compared to the relatively-stable group.

**Conclusions:**

This study shows the difference in demographics and geriatric conditions (such as SPPB) through the examination of longitudinal trajectory groups of disability in community-dwelling older adults. Significant differences were also found in the amount of home time among the trajectory groups.

**Supplementary information:**

**Supplementary information** accompanies this paper at 10.1186/s12877-020-01834-y.

## Background

The number of countries facing an aging population is increasing worldwide [1, 2]. The increase in the world’s older population implicates an increase in not only the prevalence of chronic diseases, but also the burden of people with functional impairments and geriatric conditions (e.g., frailty and sarcopenia) [3–6]. Particularly, disability, which is regarded as a health-related condition, increases care demands and socioeconomic burdens for both families and communities [7–9].

The onset of disability varies substantially among individuals with similar chronological ages [10–13]. Studies have shown that differences exist among individuals in terms of presence of multimorbidity, frailty, and disability. Evidence also suggests that disability can be prevented or delayed when accompanied by appropriate multifactorial interventions to remove risk factors and improve functioning, making disability one of the most important outcome measures in studies targeting older populations [14].

The conventional method of assessment for disability focuses on the severity of the disability at the initial diagnosis; however, this does not reflect the individuality and time course of the disability. The trajectory method of assessment focuses on the changes in the later years of life [15]. Previous studies have shown different trajectory models based on mood, physical activity, and disability in the later years of life [8, 15–17]. However, the trajectory and time course of various disabilities are still not well understood. Although some studies show trajectories and subsequent mortality with disability [15], other studies demonstrate that not all older individuals with disability in the community end up being institutionalized in chronic hospitals or other long-term care facilities [16–18]. It is unclear whether an older individual identifying with a disability has a high probability of readmission or long-term institutionalization. Furthermore, only a few studies have focused on the dynamic trajectories of disability in community-dwelling older adults [18].

Previously, trajectories of disability were not researched in relation to patient-centered outcomes. Patient-centered outcomes are the result of a healthcare system that prioritizes a patient’s needs in conjunction with the healthcare professional’s medical expertise. It focuses on health status that is meaningful to patients such as quality of life, functional status, and independent living [19–21]. In recent studies, “home time” has been proposed as a patient-centered measure relevant to the quality of life for older people [22–24]. Home time, meaning the number of days alive and spent at home, comes from the concept of patients’ wanting to maximize the number of days they can be at home rather than in hospital or nursing facilities at the end of their life. Home time focuses on priority values and purposes that are important to older patients or their families, and shows its relationship with self-rated health, mobility, self-care difficulties, and limited social activity [22–24].

The objective of this study is to explore the following: (1) how disability is divided by the trajectory method in relation to time-sequenced data in a longitudinal cohort, (2) whether the demographic and geriatric conditions differ among the trajectory groups, and (3) whether home time, a patient-centered outcome, is differentiated by the trajectory groups.

## Methods

### Study design and sample

Records from the Aging Study of Pyeongchang Rural Area (ASPRA) were analyzed. This population-based, prospective cohort study has been established to analyze aging-related changes and major health outcomes of the older population, as part of an academic-public health collaborative model. The details of this study are described elsewhere [25]. To summarize, older Korean adults in Pyeongchang-gun who met the required criteria were enrolled beginning in November 2014. The inclusion criteria of the ASPRA cohort included: (1) being aged ≥65 years; (2) being registered in the National Healthcare Service; (3) being ambulatory with or without an assistive device; (4) living at home; and (5) being able to provide informed consent. Those who were living in a nursing home, hospitalized, or bed-ridden and receiving nursing-home-level care at the time of enrollment were excluded [25]. The cohort had a participation rate of more than 90%. A baseline study on the ASPRA population showed that demographic characteristics in this population were in accordance with those of nationwide rural-dwelling older adults [25]. The Institutional Review board of Asan Medical Center, Seoul, Korea, approved the protocol for this study (IRB No. 2015-0673).

### Assessment of disability

Trained nurses assessed disability and other geriatric conditions utilizing standardized instruments every year [7]. Disability was assessed according to a 7-item activity of daily living scale (ADL; bathing, continence, dressing, eating, toileting, transferring, and washing face and hands) [5, 26], or a 10-item instrumental activity of daily living scale (IADL; food preparation, household chores, going out short distance, grooming, handling finances, laundry, managing own medications, shopping, transportation, and using a telephone) [5, 27, 28]. Disability was defined as being dependent in more than one domain in ADL and IADL. The severity of disability was conventionally operationalized into three groups: no dependency (disabled domain: 0), mild disability (disabled domain: 1), and severe disability (disabled domain: 2 or more) [29, 30].

### Assessment of geriatric conditions

Participants’ baseline demographic factors (e.g., age, sex, education (in years), living alone, and medical aid) were further examined. Physician-diagnosed chronic diseases, including angina, arthritis, asthma, cancer, chronic lung disease, heart failure, diabetes mellitus, heart attack, hypertension, kidney disease, and stroke were identified [5]. Cognitive function was assessed by the Korean version of the Mini Mental State Examination-Dementia Screening [MMSE-DS; ranged from 0 (severe cognitive impairment) to 30 (no problem) [31]. Mood status was examined by the Korean version of the center for Epidemiological Studies Depression scale [CES-D; ranged from 0 (not depressed) to 60 (severely depressed)] [32]. Nutritional status was assessed by a Mini Nutritional Assessment-Short Form [MNA-SF; ranged from 0 (malnutrition) to 14 (well-nourished)] [33]. Physical function was measured using the Short Physical Performance Battery [SPPB; ranged from 0 (worst performance) to 12 (best performance)] that covered chair stand, standing balance, and gait speed [34, 35]. The Korean version of a 5-item FRAIL scale was administered to screen frailty status [36]. Participants were interviewed concerning their history of falls in the past year.

### Calculation of home time

Registered nurses assessed the participants’ hospital use, visits to the emergency room, and institutionalization period every three months. Home time was calculated to be 365 days excluding the dates sent from the hospital and healthcare facilities [22, 23].

### Statistical analysis

To identify differences in home time among groups, a one-way analysis of variance (ANOVA) test was utilized for home time, which is a numeric variable. Regarding the categorical variables within the study, the difference among the variables was examined by employing a chi-square test. In order to examine the statistical association between home time and disability group, a Poisson regression model was applied. We estimated the incidence rate ratio (IRR) and 95% confidence intervals (CI) for home time with a Poisson regression model, adjusted for sex and age in the trajectory group [37]. In the conventional group, the year of measure was additionally adjusted.

Based on the discrepancy of the results, separate trajectories were identified according to the severity of disability using the Proc Traj procedure in SAS 9.4 [38]. The groups were divided according to the following criteria: (a) the lowest value in Bayesian Information Criteria (BIC), (b) the average posterior probability of group assignment (≥0.7), and (c) group size such that no less than 5% of the study sample were assigned to one trajectory group [39]. These analyses were performed with the 3.5.3 version in R. Two-sided *P* values of < .05 were considered statistically significant.

## Results

### Total candidates and characteristics

**Fig. 1:**
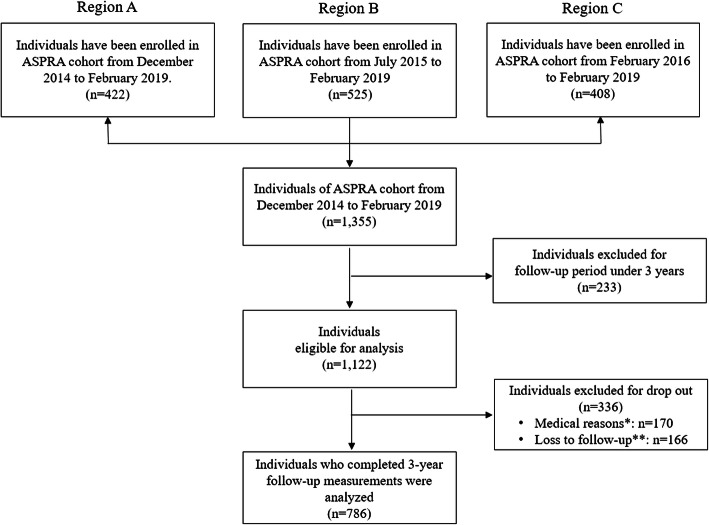
Participant selection flow. * Among the 170 participants who dropped out for medical reasons, 35 participants (20.6%) had died, 103 participants (60.6%) were admitted to nursing homes due to deterioration of health, and 32 participants (18.8%) had moved or were withdrawn due to health problems. ** Among the 166 participants who dropped out due to follow-up loss, 53 participants (31.9%) moved due to other problems (except for health), 89 participants (53.6%) declined to participate, and 24 participants (14.5%) had lost contact

Of the 1355 participants who received usual care in public health settings, those participants who had a follow-up period of less than three years were excluded. Among individuals, 233 people were excluded because the follow-up period was less than three years. By then, 336 participants dropped out due to either medical reasons (*n* = 170) or follow-up loss (*n* = 166). Among the medical reasons, 35 participants had died, 103 were admitted to nursing homes, and 32 moved out due to health problems. Of the 166 participants with follow-up loss, 53 moved out due to other problems, 89 declined to participate, and 24 had lost contact. Finally, 786 participants who completed routine measurements for three years were analyzed in this study (Fig. 1). For participants with a follow-up period longer than three years, the baseline point was defined as the first measurement after enrollment.

Participants’ baseline demographic factors including age, sex, education (in years), living alone (or not), and medical aid (or not) were examined according to total participants based on Fig. 1 (Table 1). Geriatric conditions such as number of comorbidities, MMSE-DS, number of regular medications, FRAIL scale, SPPB score, CES-D score, MNA-SF score, and the number of falls were included. The average age was 73.3 years (SD: 5.8), and the percentage of females was 52.7% of the total. The average education (in years) was 5.2 years (SD: 3.3), and 15.8% of the total were living alone. Among geriatric conditions, the baseline of the SPPB and MMSE-SD score was 8.8 (SD: 2.8) and 25.7 (SD: 3.9), respectively, each at baseline.

**Table 1:** Demographic characteristics and geriatric conditions by total participants

Variable		Total (*n* = 786)
Demographic characteristics	Age	
	*mean (SD)*	73.3 (5.8)
	Sex	
	female *no*. (%)	414 (52.7%)
	Education year	
	*mean (SD)*	5.2 (3.3)
	Living alone	
	*no*. (%)	124 (15.8%)
	Medical aid	
	*no*. (%)	23 (2.9%)
Severity of disability		
Number of ADL domains, *mean (SD)*	1st year	0.2 (0.6)
	3rd year	0.4 (0.8)
Number of IADL domains, *mean (SD)*	1st year	0.7 (1.5)
	3rd year	1.0 (2.1)
Geriatric conditions		
Number of comorbidities, *mean (SD)*	1st year	1.3 (1.0)
	3rd year	1.7 (1.2)
SPPB score, *mean (SD)*	1st year	8.8 (2.8)
	3rd year	9.3 (2.9)
MMSE-DS score, *mean (SD)*	1st year	25.7 (3.9)
	3rd year	25.0 (4.4)
CES-D score, *mean (SD)*	1st year	6.8 (8.6)
	3rd year	6.8 (9.1)
MNA-SF score, *mean (SD)*	1st year	12 (1.9)
	3rd year	12.2 (1.9)
Number of regular medications, *mean (SD)*	1st year	2.6 (2.6)
	3rd year	2.6 (2.5)
FRAIL scale, *mean (SD)*	1st year	1.2 (1.2)
	3rd year	1.3 (1.2)
Number of falls (for 12 months), *mean (SD)*	1st year	0.2 (0.8)
	3rd year	0.7 (4.8)

### Disability trajectories

**Fig. 2:**
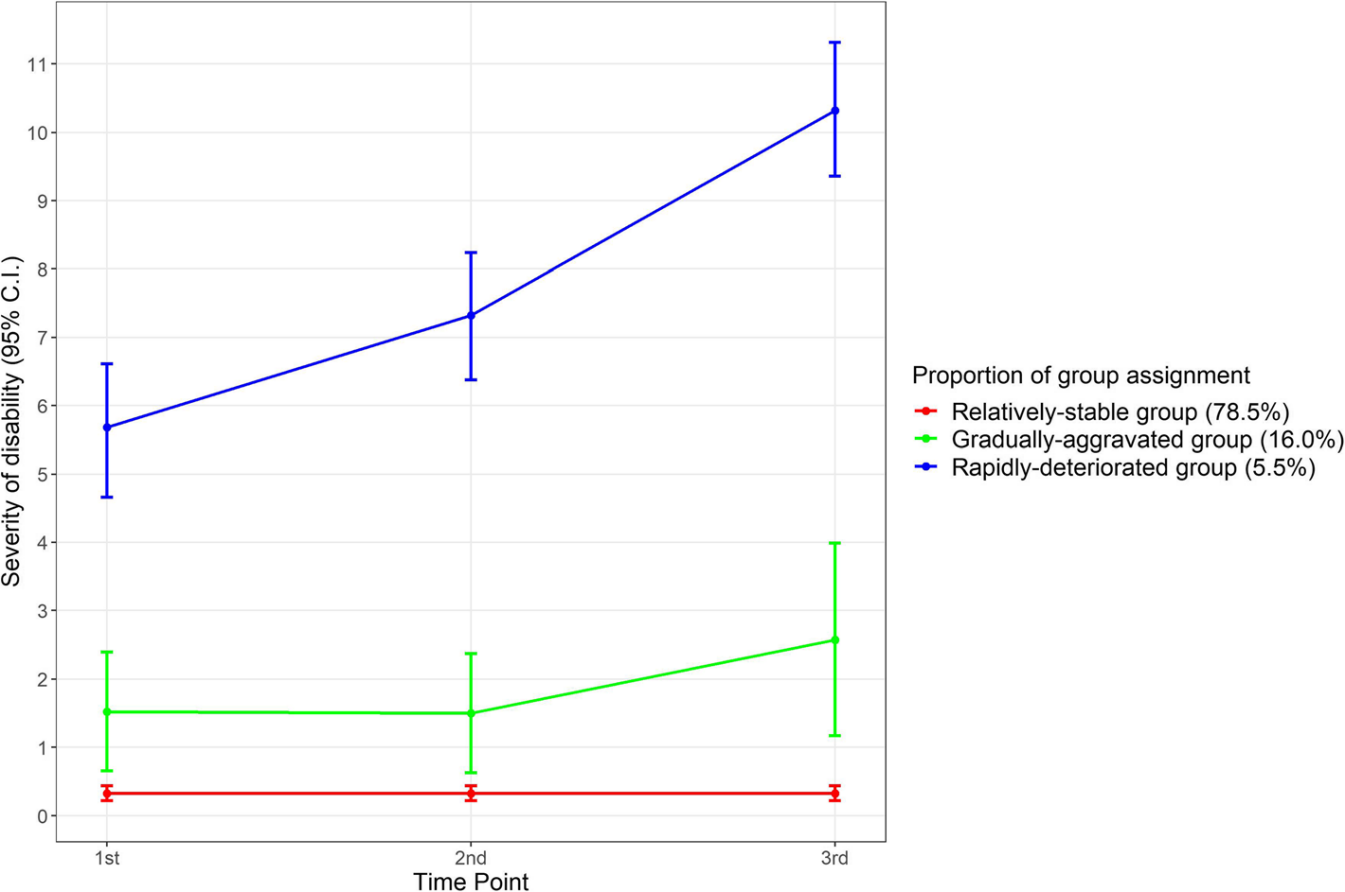
Trajectory group of disability over time (with 95% C.I., for 3-years)

Three trajectory groups were defined according to the degree of disability by the number of impaired domains from the 1st to the 3rd years (Fig. 2). The model with three trajectory groups was the best fit for our data based on BIC, considering the proportions of each group (see Table S1 on Additional file 1). The average posterior probability was assigned to each group (*p* = 0.9, 0.82, and 0.96, respectively) [39].

The “relatively-stable group” (78.5%; *n* = 617) was characterized by the lowest levels of disability. The “gradually-aggravated group” (16.0%; *n* = 126) was characterized by slightly increasing levels of disability over time. The remaining 5.5% of the population (*n* = 43) with high baseline disability that was also rapidly aggravating over time were categorized as the “rapidly-deteriorated” group (Fig. 2) [40].

### Comparisons of characteristics among trajectory groups

We looked at factors such as baseline demographic factors and geriatric condition according to the trajectory group likewise in chapter 3.1. (Table 2).

**Table 2:** Demographic characteristics and geriatric conditions by trajectory group

Variable	Relatively-stable group (***n*** = 617)	Gradually-aggravated group (***n*** = 126)	Rapidly-deteriorated group (***n*** = 43)	***P*** value*
**Demographic characteristics**				
Age				<.001
*mean (SD)*	72.1 (4.9)	76.6 (5.9)	81.1 (6.6)	
Sex				<.001
female *no*. (%)	278 (45.1%)	103 (81.8%)	33 (76.7%)	
Education year				<.001
*mean (SD)*	5.5 (3.5)	3.9 (2.1)	3.4 (1.2)	
Living alone				0.382
*no*. (%)	95 (15.4%)	19 (15.1%)	10 (23.3%)	
Medical aid				0.001
*no*. (%)	16 (2.6%)	2 (1.6%)	5 (11.6%)	
**Severity of disability**				
**Number of ADL domains,** ***mean (SD)***				
1st year	0.1 (0.2)	0.3 (0.5)	1.3 (1.7)	<.001
3rd year	0.2 (0.4)	0.6 (0.8)	2.5 (1.8)	<.001
**Number of IADL domains,** ***mean (SD)***				
1st year	0.2 (0.5)	1.7 (1.8)	4.4 (2.8)	<.001
3rd year	0.3 (0.6)	2.6 (1.8)	7.9 (1.9)	<.001
**Geriatric conditions**				
**Number of comorbidities,** ***mean (SD)***				
1st year	1.1 (1.0)	1.6 (1.0)	2.0 (1.2)	<.001
3rd year	1.6 (1.1)	2.1 (1.1)	2.4 (1.2)	<.001
**SPPB score,** ***mean (SD)***				
1st year	9.5 (2.2)	6.8 (2.9)	3.3 (2.4)	<.001
3rd year	10.2 (2.0)	7.4 (3.2)	3.1 (2.2)	<.001
**MMSE-DS score,** ***mean (SD)***				
1st year	26.5 (3.4)	23.6 (4.0)	20.4 (4.7)	<.001
3rd year	26.1 (3.3)	21.9 (5.1)	18.1 (5.0)	<.001
**CES-D score,** ***mean (SD)***				
1st year	5.4 (7.0)	11.8 (11)	13.4 (12.6)	<.001
3rd year	4.9 (6.8)	12.7 (12.5)	17.5 (12.1)	<.001
**MNA-SF score,** ***mean (SD)***				
1st year	12.3 (1.8)	11.2 (2.2)	10.6 (2.0)	<.001
3rd year	12.5 (1.7)	11.5 (2.0)	10.5 (2.5)	<.001
**Number of regular medications,** ***mean (SD)***				
1st year	2.2 (2.4)	3.5 (2.9)	4.4 (3.8)	<.001
3rd year	2.4 (2.3)	3.0 (2.5)	4.4 (3.8)	0.001
**FRAIL scale,** ***mean (SD)***				
1st year	0.9 (1.0)	2.1 (1.1)	2.5 (1.0)	<.001
3rd year	1.0 (1.1)	2.2 (1.1)	2.7 (0.8)	<.001
**Number of falls (for 12 months),** ***mean (SD)***				
1st year	0.1 (0.6)	0.3 (0.7)	0.8 (2.0)	0.047
3rd year	0.3 (1.7)	1.6 (8.4)	3 (12.9)	0.097

^*^ The *P* value given in the table uses a chi-square test and the other variables used within the one-way ANOVA

Geriatric measurements differed significantly in the three groups, except for living alone and the number of falls in the 3rd year. In the 1st year, the relatively-stable group had a mean age of 72.1 years, 45.1% were female, the mean number of comorbidities was 1.1, the number of medications was 2.2, and the mean number of falls in the previous year was 0.1. In the rapidly-deteriorated group, mean age at 1st year was 81.1 years (which is almost nine years higher than the relatively-stable group), and 76.7% of the participants were female. This group had a mean number of 2.0 for comorbidities, 4.4 for those receiving regular medications, and 0.8 for the number of falls in the previous year.

In terms of physical performance, the SPPB score was 9.5 points in the relatively-stable group and 3.3 points in the rapidly-deteriorated group. In the 3rd year, the difference between the relatively-stable group and rapidly-deteriorated group was larger than that of the 1st year, increasing from 6.1 to 7.2, respectively.

### Comparison of home time between the conventional versus trajectory-based group

Home time decreased by an incremental degree in both the conventional and trajectory-based disability groups (Table 3). Compared to the 1st year, the trend of decreasing home time took place continuously in the 2nd and 3rd year.

**Table 3:** Home time difference according to conventional versus trajectory-based grouping of disability

Year	Conventional Disability Group				Trajectory-based Group			
	No dependency group (***n*** = 518)	Mild-dependent group (***n*** = 154)	Severe-dependent group (***n*** = 114)	***p*** value*	Relatively-stable group (***n*** = 617)	Gradually-aggravated group (***n*** = 126)	Rapidly-deteriorated group (***n*** = 43)	***p*** value*
Home time (days)**, mean (SD)								
1st year	352.2 (14.3)	348.0 (22.3)	343.3 (23.1)	<.001	351.6 (14.9)	346.0 (19.7)	339.9 (37.0)	0.003
2nd year	352.0 (14.1)	347.7 (22.2)	342.9 (22.8)	<.001	351.4 (14.7)	345.7 (19.5)	339.5 (36.4)	0.002
3rd year	350.3 (17.6)	348.2 (22.2)	344.8 (20.4)	0.025	350.3 (17.6)	345.5 (17.5)	341.8 (34.6)	0.009

^*^ The *p* value given in the table uses the one-way ANOVA

In the 1st year, the home time of the severe group was shorter by 8.9 days (352.2 days–343.3 days) compared to the no dependency group by conventional grouping. In contrast, the rapidly-deteriorated group had 11.7 days fewer (351.6 days–339.9 days) home time than the relatively-stable group by trajectory-based grouping in the 1st year.

In the 3rd year, the home time of the severe group was shorter by 5.5 days (350.3 days–344.8 days) compared to the no dependency group by conventional grouping. By trajectory-based grouping, the rapidly-deteriorated group stayed 8.5 fewer days in their home than in the relatively-stable group (350.3 days–341.8 days, a 2.43% decrease).

### Incidence rate ratio for home time according to conventional versus trajectory-based grouping of disability

After recognizing the differences in home time decrements by definitions of disability phenotype (Table 2), regression models were employed to adjust for demographic factors, including age and sex, in these observations. Additionally, the year of measurement was adjusted in the conventional group since the trajectory-based definition already took into account time sequence. In the statistical model with adjusted variables, significant differences of home time between the conventional based and trajectory-based definitions were observed in the univariate analysis (see Table S2 in Additional file 1).

**Fig. 3:**
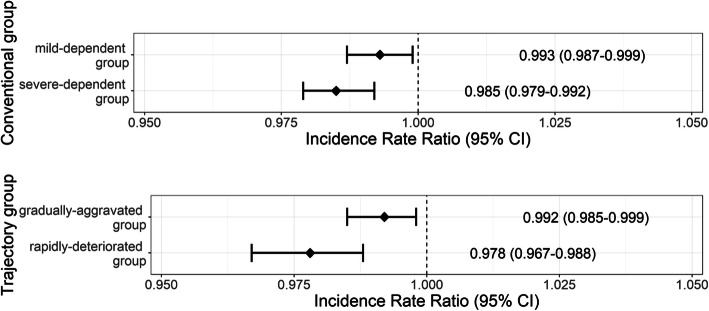
Forest plot of the incidence rate ratio for conventional versus trajectory group of disability. *The analysis of the trajectory group was adjusted for sex and age. The conventional group was additionally adjusted for the year of measurement. ** The reference value of the conventional group is the ‘no dependency group’ and the reference value of the trajectory group is the ‘relatively-stable group’

The IRR for home time in the conventional groups and trajectory groups is shown in Fig. 3. Home time in the mild dependent group (IRR = 0.993; 95% CI, 0.987–0.999) was shorter than the reference group (no dependency group) by conventional grouping. Similarly, the severe-dependent group had shorter home time (IRR = 0.985; 95% CI, 0.979–0.992) compared to the no dependency group.

In the trajectory-based group, the home time of the gradually-aggravated group was shorter (IRR =0.992; 95% CI, 0.985–0.999) compared to the relatively-stable group. Similarly, the rapidly-deteriorated group had shorter home time (IRR =0.978; 95% CI, 0.967–0.988) compared to the relatively-stable group.

### Incidence rate ratio of subgroup for home time according to trajectory-based grouping of disability

We also conducted subgroup analysis according to age and sex, respectively. In the case of age group, we divided age group criteria into (1) 65–74 years, and (2) 75 years or older based on [41]. According to our findings, home time in the female group was lower in the gradually-aggravated group and rapidly-deteriorated group than in the relatively-stable group (IRR, 0.989 and 0.967). However, in the male group, there were no significant results for either classification of disability and home time.

In the case of the age group, the IRR for home time in the 65–74 year age group was 0.915 in the rapidly-deteriorated group compared to the relatively-stable group. The portion of the rapidly-deteriorated group was just 1.7% of the total, however. In the 75 years or older group, the disability classification was proper. But it was not statistically significant with regard to home time (see Table S3, Fig. S1, and S2).

## Discussion

Disability is a major determinant of quality of life in older adults. In the present study, different trajectory groups were categorized according to the severity of disability over time. The conventional method of identifying disability shows only a snapshot of disability status and individual disability components. Therefore, the trajectory groups of disability that we identified demonstrated a more integrated approach toward defining disability.

The major finding of this study is that three trajectory groups with different severities of disability were confirmed in community-dwelling older adults. The three trajectory groups were divided into the following: a relatively-stable group (78.5%), a gradually-aggravated group (16.0%), and a rapidly-deteriorated group (5.5%). Previous studies had shown trajectory grouping using the number of disabilities in patients with underlying diseases such as cancer. In a study with cancer patients, the percentage of the severe trajectory group was 21.2% prior to receiving cancer treatment [42]. Our study is unique in that we show the percentage of the severe disability group (rapidly-deteriorated group) to be around 5.5% in relatively healthy older adults living in rural communities. Our data may serve as a basis for future reference in disability studies of the general older populations.

Another finding is that there were differences in the demographic characteristics and geriatric conditions among the different trajectory groups. Most of the variables of the demographic and geriatric conditions were significantly different trajectory groups, except for the number of falls and living alone status. We confirm that the age increased, and the years of education decreased from the relatively-stable to deteriorated group. What stands out most from this study is the change in SPPB. It is well known that SPPB is an important variable for older adults in addition to the FRAIL scale and MMSE-DS score [43]. Our results show that in the 3rd year, the SPPB score was 10.2 points (SD: 2.0) in the relatively-stable group and 3.1 points (SD: 2.2) in the rapidly-deteriorated (more severe) group. In addition to a statistical difference, the numerical difference shows that there was a difference of more than three times between the relatively-stable group and the rapidly-deteriorated group. From this result, we recommend that a comprehensive geriatric assessment in clinical settings be performed, if available, in order to measure physical performance such as SPPB.

Furthermore, our study contributes to the literature by showing that the trajectory method can maximize the difference in home time compared to the conventional method. We showed that home time decreased more over time, as the disability type was severe at initial diagnosis and the increasing levels of disability were rapid. In the 2nd and 3rd year follow-up, the decrease in home time was smaller than in the 1st year, but the home time of the trajectory groups were still reduced compared to the conventional groups, and this difference was statistically significant. We found that the trajectory method decreased 3.33% in the rapidly-deteriorated group compared to the stable group. In the conventional case, the severe-dependent group decreased by 2.53% compared to the non-dependent group.

The rapidly-deteriorated group had shorter home time (IRR = 0.978; 95% CI, 0.967–0.988) compared to the relatively-stable group by trajectory method. This result was shorter than the severe-dependent group of the conventional method (IRR = 0.985; 95% CI, 0.979–0.992).

Considering subgroup, home time in the female group was lower in the gradually-aggravated and rapidly-deteriorated group than in the relatively-stable group (IRR, 0.989 and 0.967), but males and other age groups were not significant in terms of disability classification or home time reduction.

Finally, our results can inform public health professionals developing care models to detect trajectories of disability and build individualized intervention or rehabilitation programs and health policies based on the trajectories, for older, vulnerable populations.

The strengths of this study are that the enrollment rate was 90% and based on an aging cohort derived from an academic-public health collaborative model. We obtained consistent data based on internationally validated geriatric assessment tools and, therefore, the results reflect real world data. Although our data is based on rural communities where some proportions of individuals have low education and are engaged in agriculture, it is a population-based cohort and the sociodemographic characteristics were similar to those of the representative Korean national data.

This study has several limitations. Among the 1122 eligible participants, 166 people (15%) were lost to follow-up. This may be a limitation in constructing the trajectory model, however, this 15% follow- up loss was over the three years of analysis. Therefore, loss to follow-up occurred around 5% per year, which is less than the general percentage of population migration. Second, there may be a recall bias in home time. The participants may not fully recall their hospital or emergency visits in the previous years. In order to overcome this limitation, we obtained information from Community Health Posts in Pyeonchang run by the National Healthcare Service for information if the participants were not fully aware of their hospital use in the past. Therefore, we attempted to minimize recall bias. Third, there was a relatively short follow-up term. The cohort was a three-year follow-up study and, therefore, there is a need for individuals to be examined over longer periods of time. Lastly, it is difficult to capture the short- and medium-term changes in disability lasting less than a year by the methods we used. Gill et al. have suggested that mechanisms underlying the different subtypes are likely to differ. While the presence of physical frailty increased the likelihood of developing long-term, recurrent, and unstable disability, it only had a modest effect on developing transient and short-term disability [44].

## Conclusions

A longitudinal trajectory method was used to apply the time trend of disability to community-dwelling older adults. We verify that the demographical and clinical indexes are different according to the trajectory grouping, and the significant effect of the trajectory method on home time was also examined. Our observations provide public health professionals and policy makers with valuable information in order to set priorities for policy making and intervention.

## Supplementary information

**Additional file 1: Supplementary Table 1.** Tabulated BIC’s and 2 Δ BIC. **Supplementary Table 2.** Result of poisson regression for home time. **Supplementary Table 3.** Result of sub-group analysis of poisson regression for home time. **Supplementary Figure 1.** Trajectory grouping in under 74-years old and over 75-years old. **Supplementary Figure 2.** Trajectory grouping in male and female

## Data Availability

The datasets used are available from the corresponding author on reasonable request.

## Abbreviations

ANOVA: Analysis of Variance
BIC: Bayesian information criterion
SPPB: Short Physical Performance Battery
ADL: Activity of Daily Living
IADL: Instrumental Activity of Daily Living
MMSE-DS: Mini Mental State Examination-Dementia Screening
CES-D: Center for Epidemiological Studies Depression scale
MNA-SF: Mini Nutritional Assessment-Short Form
ASPRA: Aging Study of Pyeongchang Rural Area
IRR: Incidence Rate Ratio
