# Supplementary material for: Longitudinal trajectory of disability in community-dwelling older adults: An observational cohort study in South Korea
Source: BMC Geriatr. 2020 Oct 28;20:430. doi: 10.1186/s12877-020-01834-y (PMC7594294; doi:10.1186/s12877-020-01834-y)

**Additional file 1**

**Longitudinal trajectory of disability in community-dwelling older adults:**

**An observational cohort study in South Korea**

**Table and Figure of Contents**

| **Supplementary Table 1.** | Tabulated BIC’s and 2$\Delta$BIC |
| --- | --- |
| **Supplementary Table 2.** | Result of poisson regression for home time |
| **Supplementary Table 3**. | Result of sub-group analysis of poisson regression for home time |
| **Supplementary Figure 1.** | Trajectory grouping in under 74-years old and over 75-years old |
| **Supplementary Figure 2**. | Trajectory grouping in male and female |

**Table S1**. Tabulated BIC’s and 2$\Delta BIC$

| Number of groups | $\mathrm{BIC}_{complex}$ | $\mathrm{BIC}_{null}$ | 2$\Delta BIC*$ | Proportion of group assignment | | | | |
| --- | --- | --- | --- | --- | --- | --- | --- | --- |
|  |  |  |  | Group1 | Group2 | Group3 | Group4 | Group5 |
| 1 | -3395.43 | -3396.53 | 2.2 | 100 | - | - | - | - |
| 2 | -3139.61 | -3142.90 | 6.58 | 91.6 | 8.4 | - | - | - |
| 3 | -3103.65 | -3109.14 | 10.98 | 78.5 | 16.0 | 5.5 | - | - |
| 4 | -3092.54 | -3100.23 | 15.38 | 35.7 | 55.5 | 7.4 | 1.4 | - |
| 5 | -3086.24 | -3096.13 | 19.78 | 0.0 | 88.2 | 7.0 | 3.3 | 1.5 |

*∆BIC = $\mathrm{BIC}_{complex}$- $\mathrm{BIC}_{null}$. If 0< 2$\Delta BIC<2$, then there are no difference (between two models). Else if 2< 2$\Delta BIC<6$, then there is positive difference. Else if 6< 2$\Delta BIC<10$, then there is strong difference. Else if 10< 2$\Delta BIC,$ then there is very strong difference (between two models).

**Table S2**. Result of poisson regression for home time

| Category | Model | IRR** | Lower Limit  (2.5%) | Upper Limit  (97.5%) | Group*** |
| --- | --- | --- | --- | --- | --- |
| Unadjusted | Conventional group | 0.99 | 0.984 | 0.996 | mild-dependent group |
|  |  | 0.978 | 0.972 | 0.984 | severe-dependent group |
|  | Trajectory group | 0.985 | 0.979 | 0.99 | gradually-aggravated group |
|  |  | 0.969 | 0.96 | 0.979 | rapidly-deteriorated group |
| Adjusted* | Conventional group | 0.993 | 0.987 | 0.999 | mild-dependent group |
|  |  | 0.985 | 0.979 | 0.992 | severe-dependent group |
|  | Trajectory group | 0.992 | 0.985 | 0.998 | gradually-aggravated group |
|  |  | 0.978 | 0.967 | 0.988 | rapidly-deteriorated group |

* The analysis of trajectory group was adjusted with gender and age. And that of conventional group was further corrected with the year of measure

**IRR, Incidence Rate Ratio

*** The reference value of conventional group is ‘no dependency group’, and the reference value of trajectory group is ‘relatively-stable group’

**Table S3**. Result of sub-group analysis of poisson regression for home time

| Category | Model | IRR** | Lower Limit  (2.5%) | Upper Limit  (97.5%) | Group*** |
| --- | --- | --- | --- | --- | --- |
| Gender | Male | 1.007 | 1.001 | 1.014 | gradually-aggravated group  in male group |
|  |  | 1.004 | 0.985 | 1.022 | rapidly-deteriorated group  in male group |
|  | Female | 0.989 | 0.983 | 0.996 | gradually-aggravated group  in female group |
|  |  | 0.967 | 0.955 | 0.979 | rapidly-deteriorated group  in female group |
| Age  Group | 65-74 | 1.009 | 1.003 | 1.015 | gradually-aggravated group  in from 65 to 74 group |
|  |  | 0.915 | 0.894 | 0.936 | rapidly-deteriorated group  in from 65 to 74 group |
|  | Over 75 | 0.995 | 0.986 | 1.005 | gradually-aggravated group  in over 75 group |
|  |  | 0.998 | 0.985 | 1.012 | rapidly-deteriorated group  in over 75 group |

* The analysis of trajectory group was adjusted with gender and age. And that of conventional group was further corrected with the year of measure

**IRR, Incidence Rate Ratio

**Figure S1**. Trajectory grouping in under 74-years old (high) and over 75-years old (bottom)


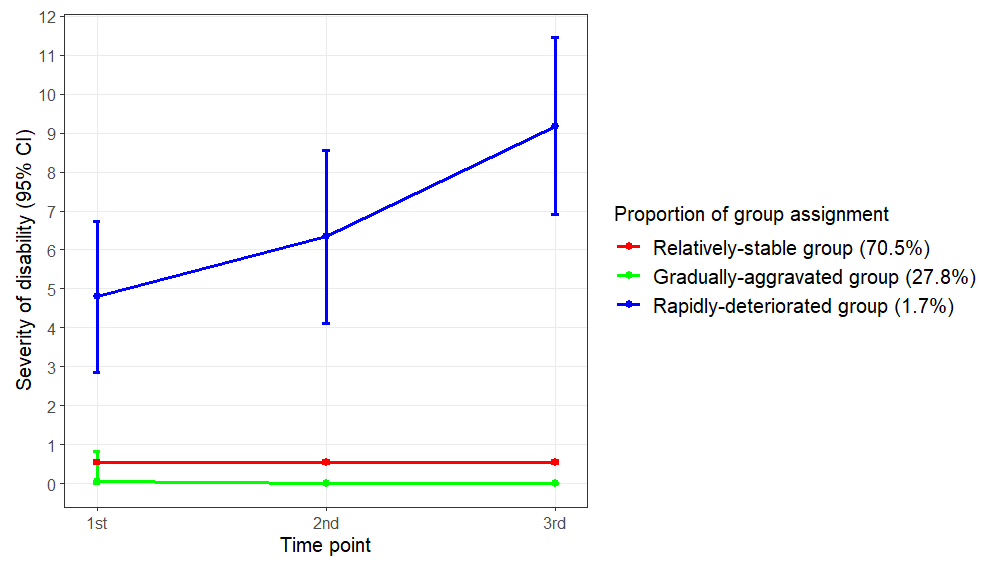


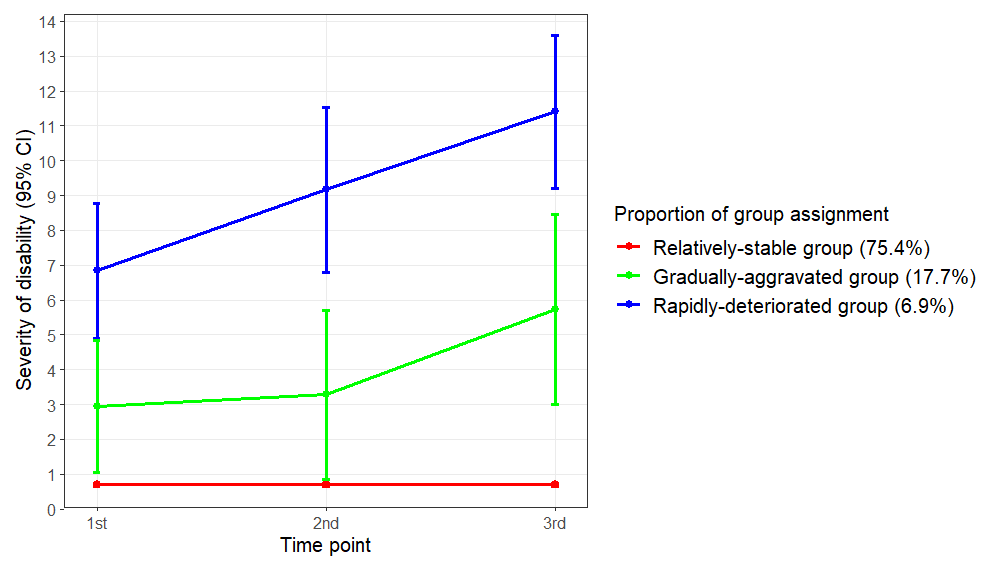


**Figure S2**. Trajectory grouping in male (high) and female (bottom)


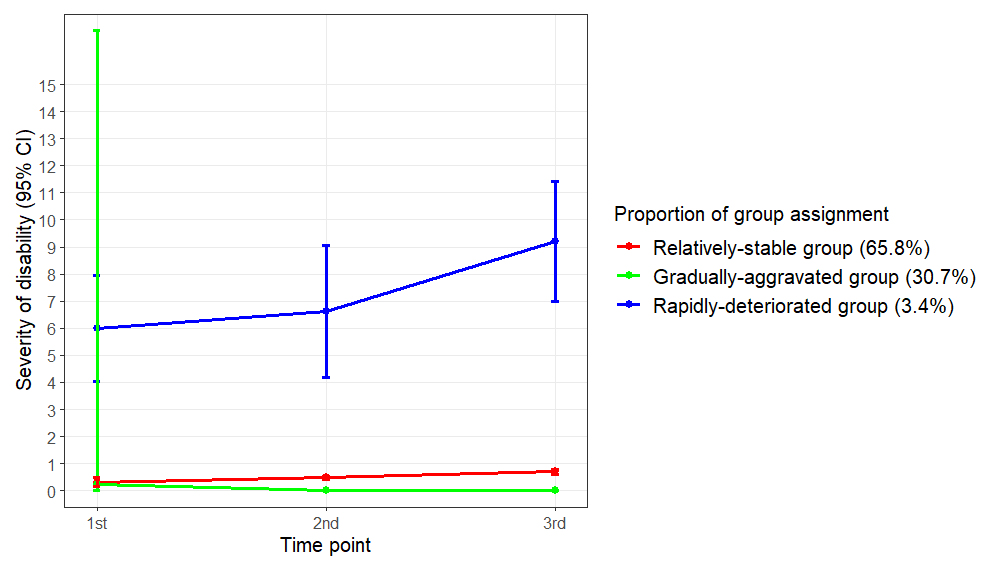


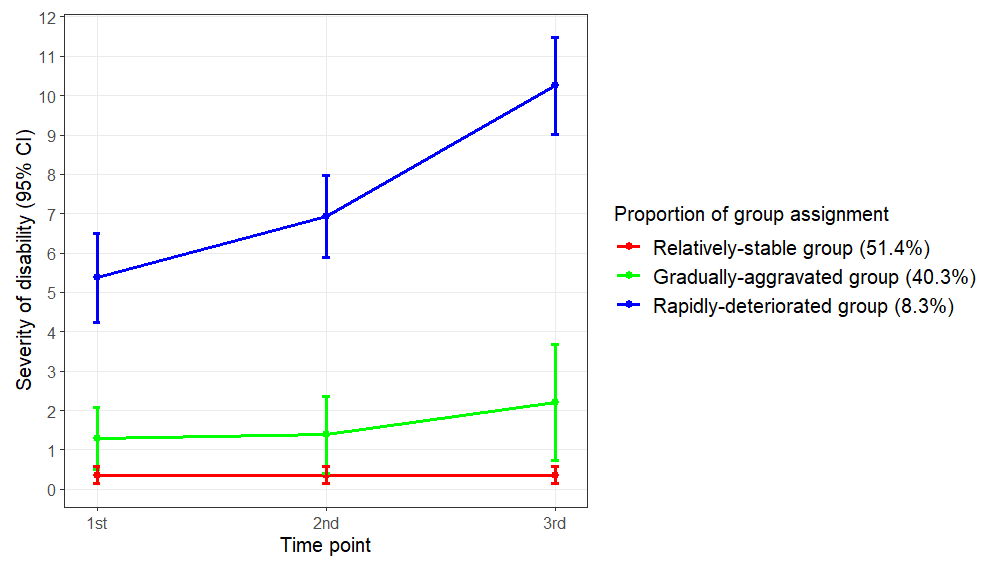

Supplement: Supplementary file 1 — Additional file 1: Supplementary Table 1. Tabulated BIC’s and 2 Δ BIC. Supplementary Table 2. Result of poisson regression for home time. Supplementary Table 3. Result of sub-group analysis of poisson regression for home time. Supplementary Figure 1. Trajectory grouping in under 74-years old and over 75-years old. Supplementary Figure 2. Trajectory grouping in male and female [file 12877_2020_1834_MOESM1_ESM.docx]
